# Theoretical Analysis of Critical Flowable Physical Gel Cross-Linked by Metal Ions and Polyacrylamide-Derivative Associating Polymers Containing Imidazole Groups

**DOI:** 10.3390/polym9070256

**Published:** 2017-06-29

**Authors:** Hiroto Ozaki, Tetsuharu Narita, Tsuyoshi Koga, Tsutomu Indei

**Affiliations:** 1 Department of Polymer Chemistry, Graduate School of Engineering, Kyoto University, Katsura, Kyoto 615-8510, Japan; ozaki@phys.polym.kyoto-u.ac.jp (H.O.); tkoga@phys.polym.kyoto-u.ac.jp (T.K.); 2 Laboratoire Sciences et Ingénierie de la Matière Molle, PSL Research University, UPMC Univ Paris 06, ESPCI Paris, CNRS, 10 rue Vauquelin, 75231 Paris CEDEX 05, France; tetsuharu.narita@espci.fr; 3 Global Station for Soft Matter, Global Institution for Collaborative Research and Education, Hokkaido University, Sapporo 001-0021, Japan; 4 Department of Chemical and Biological Engineering, and Center for Molecular Study of Condensed Soft Matter, Illinois Institute of Technology, 3440 S. Dearborn Street, Suite 150, Chicago, IL 60616, USA

**Keywords:** associating polymer solution, physical gelation, critical gel, hydrogel, linear rheology, microrheology

## Abstract

When the polymer chains are cross-linked by physical bonds having a finite lifetime, the relaxation time and viscosity do not diverge at the gel point though percolation occurs. These undivergent quantities are related to the finite-sized “largest relaxed cluster,” which can relax before it breaks. Its size is the key rheological parameter characterizing of the critical physical gels. In order to evaluate this characteristic size, we propose here a generalized phenomenological model for the viscoelasticity of critical physical gels. We apply the theory to the previously reported experimental result for the physical gel consisting of polyacrylamide-derivative associating polymers containing imidazole groups cross-linked by coordination bonds with Ni ions. We successfully estimate the size of the largest relaxed cluster and the fractal dimension. The size is in good agreement with that estimated from the mean-square displacement of probe particles at the gel point by microrheological measurement. We also compare this system with the poly(vinyl alcohol) hydrogel cross-linked by borate ions, and show that the difference in the cluster structures is originating from the differences of precursor chain properties such as overlap concentration and radius of gyration and of the cross-linking states in these systems.

## 1. Introduction

In a polymer solution in which polymer chains can be cross-linked to each other, a three-dimensional network structure, or gel, is formed as the extent of cross-linking increases [1,2,3]. Such a specific condition where the gel appears is called the gel point or the gelation point [1,2,3,4,5,6]. Recently, we have comprehensively studied dynamic-mechanical and structural properties of polyacrylamide-derivative associating polymers containing imidazole groups (PAAmVIm) across the gelation point using diffusing-wave spectroscopy (DWS) microrheology, macrorheology, and dynamic light scattering (DLS) [7]. This physical gel is cross-linked by coordination bonds between imidazole groups on the PAAmVIm chains and Ni ions added into the PAAmVIm solution. The solvent is water, which behaves as a good solvent for the PAAmVIm chain. The gelation is physical in a sense that not only the cross-links are due to noncovalent bonding but the resulting gel flows in a typical observation time (e.g., on the order of seconds). This kind of physical gels formed by metal–ligand bonds shows controllable viscoelastic properties by changing the metal ion, the ligand, and pH [8,9]. Those viscoelastic properties and the relaxation mechanisms have been systematically studied as a model system of physical gels [10,11,12]. In this paper, we theoretically analyze the PAAmVIm–Ni physical hydrogels at the gel point from a rheological viewpoint.

It is widely known among rheologists that the storage modulus $G^{\prime }\left(\omega \right)$ and the loss modulus $G^{\prime \prime }\left(\omega \right)$ at a gel point show a power-law behavior $G^{\prime }\left(\omega \right)\propto G^{\prime \prime }\left(\omega \right)\propto \omega^{n}$ with the same exponent *n* in a wide range of frequency ω [13]. Hereafter, we call *n* the viscoelastic exponent. This phenomenon was first observed and comprehensively studied by Winter and Chambon (WC) for chemical gels [13], and later several permanent physical gels were also shown to obey the same rule [14,15,16,17]. (If a gel is cross-linked by noncovalent bonding but does *not* flow in a typical experimental time scale, we call it a permanent physical gel.). The WC law of gelation is a consequence of a self-similar relaxation spectrum and fractal structure of the material where the characteristic time and length are “infinite” or out of the experimental window [13,18,19]. The WC law was also successfully observed by some of the authors for a typical impermanent, or temporal, physical gel, i.e., poly(vinyl alcohol) (PVA) cross-linked by borate ions [20]. By using the DWS-microrheology, both the flow behavior (terminal zone) and the power-law behavior (WC law) were observed in $G^{*}\left(\omega \right)$ of the PVA–borax solution at the physical gel point. This is a consequence of the fact that the percolated network has finite lifetime cross-links. The PAAmVIm–Ni physical gel, which we study in this paper, is weaker than the PVA–borax physical gel in a sense that the bond strength connecting the polymer chains is weaker, and, as a result, the frequency regime where the WC law appears is expected to be narrower. Nevertheless, in our previous study, we could observe both the flow and power-law behaviors in $G^{*}\left(\omega \right)$ of the PAAmVIm–Ni solution at the physical gel point by using the DWS-microrheology [7] because this technique allows us to measure the linear viscoelastic properties of a sample at frequencies ranging from approximately $10^{1}\mathrm{Hz}$ to $10^{5}\mathrm{Hz}$.

Our aim in this paper is to estimate the size and the relaxation time of the largest relaxed cluster (see Section 3 for details) that characterizes the rheology and the structure of the PAAmVIm–Ni physical hydrogel at the gel point. For this purpose, we use a phenomenological model to describe the linear viscoelasticity of such critical physical gels that was previously introduced for the analysis of the PVA–borax critical physical gels [20]. We discuss the cluster structure and the rheological behavior by comparing the present results with those for the PVA–borax gels (Section 4).

## 2. Theory of Critical Physical Gelation

In this section, we explain the phenomenological theory that describes the linear viscoelasticity of critical physical gels. This is based on the theory that some of the authors introduced previously, but is generalized so as to include internal dynamics of the precursor chain in the good solvent condition.

### 2.1. Size of Clusters

Firstly, we consider a pregel regime. According to the blob picture [2], a precursor chain in the good solvent condition is described by a Gaussian chain of blobs. If the blob size (or correlation length) is ξ and the number of blobs per chain is *N*, then the radius of gyration of a precursor chain is

(1) $$\begin{array}{c} R_{g}≅N^{1/2}\xi . \end{array}$$

The associative groups on the polymer chains can be cross-linked to each other via the cross-linkers. Thus, the clusters of various sizes formed by the chains are present in the solution. The radius of gyration of the cluster constructed by *m* chains (hereafter, referred to as the *m*-cluster) can be expressed as

(2) $$\begin{array}{c} R_{m}≅m^{1/d_{f}}R_{g}, \end{array}$$

where $d_{f}$ is the fractal dimension of the cluster. Here, we assumed that the radius of gyration of precursor chain $R_{g}$ is independent of the cross-linking state since the number of potentially cross-linkable sites per chain is small in this system (the detail is shown in Section 4). By putting Equation (1) into Equation (2), the radius of gyration of the *m*-cluster is written as

(3) $$\begin{array}{c} R_{m}≅m^{1/d_{f}}N^{1/2}\xi . \end{array}$$

With increasing the cross-linker concentration, the cluster size becomes large because the number of chains participated in the cluster increases on average. However, the lifetime of the cluster becomes short as the cluster grows in size because the larger cluster has more breakable physical cross-links in the structure. As a result, near the gel point (inside what we call the Rubinstein–Semenov regime [6,7,20,21]), clusters larger than a certain size can break before they relax into equilibrium [6]. This process defines the “largest relaxed cluster” near the gel point. Let $m_{z}$ be the number of the chains involved in such a largest cluster. Then, the size of the largest relaxed cluster, $R_{z}$, is obtained by putting $m=m_{z}$ in Equation (3), i.e.,

(4) $$\begin{array}{c} R_{z}≅m_{z}^{1/d_{f}}N^{1/2}\xi . \end{array}$$

Clusters larger than $R_{z}$ are broken before relaxing, and they do not contribute to the rheological properties [20]. Therefore, the rheological properties of transient physical gels near the gel point can be characterized by $m_{z}$, $R_{z}$, and the lifetime $\tau_{z}$, which is equal to the relaxation time of the largest relaxed cluster (discussed below).

### 2.2. Relaxation Time of Clusters

If the internal relaxation mode of the blob is neglected, there are mN relaxation modes for a single *m*-cluster composed of mN blobs. Among those relaxation modes, the first m-1 modes (i.e., j=1,⋯,m-1) describe the relaxation of a larger scale structure than the precursor chain. On the other hand, the remaining m(N-1)+1 modes (i.e., j=m,⋯,mN) describe the internal relaxation modes of the precursor chain, i.e., these modes are equivalent to the Rouse modes. Considering the self-similarity in the Brownian dynamics of the connected chains, and due to the continuity of the relaxation spectrum at the *m*th mode, the relaxation time of the *j*th mode of the *m*-cluster is given by

(5) $$\tau_{j,mN}=\{\begin{array}{c} j^{-\alpha }\tau_{m}^{\max}\;\;\left(j=1,\cdots ,m-1\right),\\ {\left(\frac{j}{m}\right)}^{-2}\tau_{R}\;\;\left(j=m,\cdots ,mN\right), \end{array}$$

where the exponent α is determined from experiments (see Section 3.3). In the first line, $\tau_{m}^{\max}$ is the longest relaxation time of the *m*-cluster: $\tau_{m}^{\max}\equiv \tau_{j=1,mN}$. In addition, in the second line, $\tau_{R}$ is the Rouse relaxation time of the precursor chain (i.e., the longest relaxation time of the chain): $\tau_{R}\equiv \tau_{j=m,mN}\left(=\tau_{m=1}^{\max}\right)$. Note that $\tau_{R}$ is proportional to $N^{2}$ but is independent of *m* (see Equation (21)). In Section 3.2, we determine $\tau_{R}$ from experimental results. Since the spectrum is continuous at the boundary j=m, we see that $\tau_{R}$ is also described as

(6) $$\begin{array}{c} \tau_{R}\left(=\tau_{j=m,mN}\right)=m^{-\alpha }\tau_{m}^{\max}. \end{array}$$

Therefore, we can rewrite Equation (5) as

(7) $$\begin{array}{cc} \tau_{j,mN} & ={\left(\frac{j}{m}\right)}^{-\Delta }\tau_{R}, \end{array}$$

where

(8) $$\begin{array}{cc} \Delta & \equiv \left\{\begin{array}{cc} \alpha & (j=1,\cdots ,m-1),\\ 2 & (j=m,\cdots ,mN). \end{array}\right. \end{array}$$

On the other hand, the shortest relaxation time of the cluster $\tau_{0}$ is given by

(9) $$\begin{array}{c} \tau_{0}\equiv \tau_{j=mN,mN}=N^{-2}\tau_{R}. \end{array}$$

Note that $\tau_{0}$ does not depend on *m* nor *N* since $\tau_{R}$ is proportional to $N^{2}$. The relaxation time of the smallest unit of the cluster does not depend on the properties of larger scale structure, i.e., the cluster size and the precursor chain length.

The longest relaxation time of the largest relaxed cluster, $\tau_{z}$, is a rheologically important quantity. This quantity is roughly comparable to the lifetime of the largest relaxed cluster because the clusters that are larger than that break before they relax [6]. Thus, $\tau_{z}$ characterizes the onset of the terminal zone in the relaxation modulus. By putting $m=m_{z}$ and j=1 in Equation (7), $\tau_{z}$ is obtained as

(10) $$\begin{array}{c} \tau_{z}=\tau_{j=1,m_{z}N}=m_{z}^{\alpha }\tau_{R}. \end{array}$$

### 2.3. Dynamic Mechanical Properties of Solution (Pregel)

We express the complex modulus originating from the *m*-clusters in the form of the generalized Maxwell model, that is,

(11) $$\begin{array}{c} G_{m}^{*}\left(\omega \right)=g_{0}\sum_{j=1}^{mN}\frac{i\omega \tau_{j,mN}}{1+i\omega \tau_{j,mN}}, \end{array}$$

where the prefactor $g_{0}$ represents the magnitude of the modulus. Substituting Equation (7) into Equation (11), we obtain

(12) $$\begin{array}{cc} G_{m}^{*}\left(\omega \right) & =g_{0}\sum_{j=1}^{mN}\frac{i\omega {\left(j/m\right)}^{-\Delta }\tau_{R}}{1+i\omega {\left(j/m\right)}^{-\Delta }\tau_{R}}. \end{array}$$

In calculating the complex modulus of the solution, the polydispersity of the cluster size must be taken into consideration. If the cluster size distribution is $N_{m}$, then the complex modulus of the solution is given as the sum of the contributions from each *m*-cluster given by Equation (12) with the weight $N_{m}$. That is,

(13) $$\begin{array}{c} G^{*}\left(\omega \right)=\sum_{m=1}^{M}N_{m}G_{m}^{*}\left(\omega \right). \end{array}$$

The upper bound of the summation M is the number of polymers composing of the largest cluster in the solution.

### 2.4. Dynamic Mechanical Properties at Physical Gel Point

Secondly, we consider the gel point. As a reference, let us first consider chemical gels [4,22]. At a chemical gel point, $m_{z}$ becomes infinitely large. In addition, the structure of the material becomes self-similar with no characteristic length, and therefore the distribution function of the cluster size obeys the power law:

(14) $$\begin{array}{c} N_{m}=N_{1}m^{-\tau_{F}}, \end{array}$$

where we neglected the low and high cutoffs for simplicity. The exponent $\tau_{F}$ is often called the Fisher exponent [4]. The Fisher exponent is related to the viscoelastic exponent *n* as [22]

(15) $$\begin{array}{c} n=\frac{\tau_{F}-1}{\alpha }. \end{array}$$

On the other hand, in the case of the transient physical gel like we are studying, the upper bound of the summation in Equation (13) is $M=m_{z}$ because the large unrelaxed clusters that are broken before they relax do not contribute to the rheological properties. Therefore, the dynamic modulus of the critical physical gel is expressed as

(16) $$\begin{array}{c} G^{*}\left(\omega \right)=\sum_{m=1}^{m_{z}}N_{m}G_{m}^{*}\left(\omega \right). \end{array}$$

By inputting Equations (12), (14), and (15) into Equation (16), it becomes

(17) $$\begin{array}{c} G^{*}\left(\omega \right)=G_{0}\sum_{m=1}^{m_{z}}m^{-(n\alpha +1)}\sum_{j=1}^{m}\frac{i\omega {\left(j/m\right)}^{-\alpha }\tau_{R}}{1+i\omega {\left(j/m\right)}^{-\alpha }\tau_{R}}, \end{array}$$

where $G_{0}\equiv g_{0}N_{1}$ is determined from experiments (see Section 3.3).

In the previous study for the PVA–borax gels, internal relaxation modes of the precursor chain were not considered [20]. Here, we employ the same assumption to make the current condition the same as that in the analysis of the PVA–borax system, and neglect these internal modes, i.e., we put N=1 in Equation (12) and in the subsequent equations. (In these equations, the symbol *N* remains. We dare to do so to make the discussion general. When the internal modes have to be considered, a value larger than 1 is put in *N* in these equations.)

**Figure 1:**
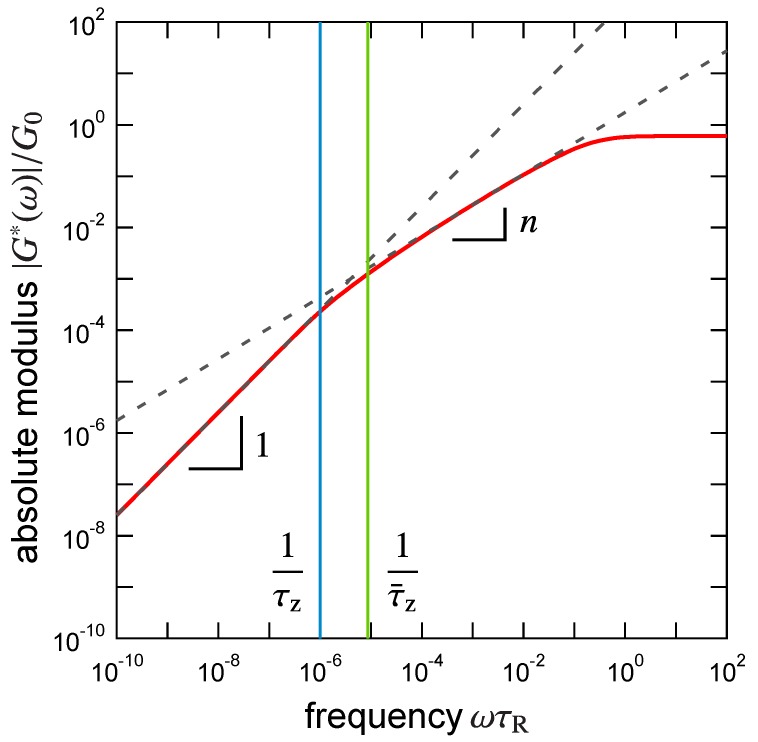
Absolute modulus (nondimensionalized by $G_{0}$) of a typical critical physical gel calculated from Equation (17). Two dashed straight lines with the exponents n(=0.6) and 1 are guides for the eyes. Blue and green vertical lines indicate reciprocals of the true longest relaxation time $1/\tau_{z}$ and the apparent relaxation time $1/{\bar{\tau }}_{z}$, respectively.

As a reference, an example of the absolute modulus derived from Equation (17) is plotted in Figure 1 for a typical set of parameter values: α=3, n=0.6, and $m_{z}=100$. In this plot, both the terminal flow behavior $|G^{*}\left(\omega \right)|∼\omega$ and the critical power-law behavior $|G^{*}\left(\omega \right)|∼\omega^{n}$ are observed in low frequency regime $\omega \ll 1/(\tau_{R}m_{z}^{\alpha })$ and in high frequency regime $1/\left(\tau_{R}m_{z}^{\alpha }\right)\ll \omega \ll 1/\tau_{R}$, respectively [20]. The reciprocal relaxation time of the largest relaxed cluster $\tau_{z}$ is comparable to the onset of the terminal flow regime (see Equation (10)). On the other hand, the “apparent” longest relaxation time is obtained as [20]

(18) $$\begin{array}{cc} {\bar{\tau }}_{z} & =\lim_{\omega \to 0}\frac{G^{\prime }\left(\omega \right)}{G^{\prime \prime }\left(\omega \right)\omega }\\ & ≃\frac{m_{z}^{\alpha }\left(n\alpha -1\right)-m_{z}^{(n-1)\alpha }\left(2\alpha -1\right)+m_{z}^{1-\alpha }\left(2-n\right)\alpha }{n\alpha -1-m_{z}^{(n-1)\alpha }\left(\alpha -1\right)+m_{z}^{1-\alpha }\left(1-n\right)\alpha }\frac{\left(1-n)(\alpha -1\right)}{\left(2-n)(2\alpha -1\right)}\tau_{R}. \end{array}$$

In the derivation, the summations over the mode *j* and the cluster size *m* in Equation (17) were approximated by the integration (see Equation (15) of Ref. [20]). This approximation is appropriate when $m_{z}\gg 1$. This apparent longest relaxation time is the weight-average relaxation time [20,23], and therefore the inequality ${\bar{\tau }}_{z}\leq \tau_{z}$ holds as we can confirm in Figure 1. The equality (${\bar{\tau }}_{z}=\tau_{z}$ ) is attained only if the cluster size distribution is monodisperse [20].

In the PAAmVIm–Ni system, the cluster size should be small due to the weak physical cross-links. Therefore, we do not use the integration approximation in calculating $G^{*}\left(\omega \right)$ when analyzing experimental results.

### 2.5. Fractal Dimension

Finally, we explain some relations between exponents obtained in Section 2.4 and the fractal dimension. Most of the relations are already known [22,24,25].

When hydrodynamic interactions between blobs are negligible (i.e., Rouse model), the friction coefficient of the *m*-cluster is given by $\zeta_{m}≅mN\zeta_{0}$, where $\zeta_{0}$ is the friction coefficient of the blob. Therefore, according to the Einstein relation [26], the diffusion coefficient of the *m*-cluster is

(19) $$\begin{array}{cc} D_{m} & ≅\frac{k_{B}T}{\zeta_{m}}≅\frac{k_{B}T}{mN\zeta_{0}}, \end{array}$$

where $k_{B}$ is the Boltzmann constant, and *T* is the absolute temperature of the solution. Thus, the longest relaxation time of the *m*-cluster can be approximately estimated as

(20) $$\begin{array}{c} \tau_{m}^{\max}≅\frac{R_{m}^{2}}{D_{m}}≅m^{\left(d_{f}+2\right)/d_{f}}\frac{\zeta_{0}\xi^{2}N^{2}}{k_{B}T}, \end{array}$$

where Equations (3) and (19) were used for the second equality. From Equations (6) and (20), the Rouse relaxation time of the chain is written as

(21) $$\begin{array}{c} \tau_{R}≅m^{-\alpha +\left(d_{f}+2\right)/d_{f}}\frac{\zeta_{0}\xi^{2}N^{2}}{k_{B}T}. \end{array}$$

Since $\tau_{R}$ should be independent of the cluster size *m*, a relation $-\alpha +\left(d_{f}+2\right)/d_{f}=0$ holds. Thus, we find a relation between the fractal dimension and the exponent α as

(22) $$\begin{array}{c} d_{f}=\frac{2}{\alpha -1}, \end{array}$$

and the Rouse relaxation time of the chain is $\tau_{R}≅\zeta_{0}\xi^{2}N^{2}/k_{B}T$ [3].

In the concentration region where the gelation occurs, the excluded-volume effect might be screened [2,25,27,28]. Let ${\bar{d}}_{f}$ be the fractal dimension in that situation. It is related to $d_{f}$ via [24,25]

(23) $$\begin{array}{c} {\bar{d}}_{f}=\frac{2d_{f}}{d_{s}+2-2d_{f}}, \end{array}$$

where $d_{s}\left(=3\right)$ is the space dimension. If the excluded-volume effect is fully screened, by replacing $d_{f}$ with ${\bar{d}}_{f}$ in Equation (22) and by using Equation (23), the fractal dimension is obtained as

(24) $$\begin{array}{c} d_{f}=\frac{d_{s}+2}{\alpha +1}. \end{array}$$

Meanwhile, from Equations (15) and (22), the relation between the viscoelastic exponent *n* and the fractal dimension $d_{f}$ is obtained as [25]

(25) $$\begin{array}{c} n=\frac{d_{s}}{d_{f}+2}, \end{array}$$

where the hyperscaling hypothesis was assumed [29]:

(26) $$\begin{array}{c} \tau_{F}=\frac{d_{s}+d_{f}}{d_{f}}. \end{array}$$

From Equation (25), the fractal dimension when the hyperscaling hypothesis is assumed, and also when the excluded volume effect is unscreened, is obtained as

(27) $$\begin{array}{c} d_{f}=\frac{d_{s}}{n}-2. \end{array}$$

As in the case where the hyperscaling hypothesis is not assumed, i.e., Equations (22) and (24), the fractal dimension when the excluded-volume effect is fully screened is obtained by replacing $d_{f}$ with ${\bar{d}}_{f}$ in Equation (27) and by using Equation (23):

(28) $$\begin{array}{c} d_{f}=\frac{\left(d_{s}-2n\right)\left(d_{s}+2\right)}{2(d_{s}-n)}. \end{array}$$

Summarizing, in determining the fractal dimension, we consider four different cases depending on whether the excluded-volume effect is unscreened or screened, and whether the hyperscaling hypothesis is assumed or not. See Table 1.

## 3. Results

In this section, we apply this theory to the critical physical gel of polyacrylamide-derivative associating polymers containing imidazole groups (PAAmVIm) physically cross-linked by transition metal ions (Ni^2+^), and determine the five unknown parameters in Equation (17): $G_{0}$, $\tau_{R}$, α, *n*, and $m_{z}$. We use the experimental results of our previous study [7]. In Ref. [7], we estimated $G^{*}\left(\omega \right)$ of this material by using microrheology based on diffusing-wave spectroscopy, and found that the Ni ion concentration where the gelation occurs (for the fixed PAAmVIm concentration $C_{p}=4\mathrm{wt}\%$) is near $C_{\mathrm{Ni}}=1.1\mathrm{mM}$. For the experimental details, see Ref. [7].

### 3.1. Viscoelastic Exponent *n* for the Dynamic Modulus

The viscoelastic exponent *n* can be experimentally determined from the loss tangent $\tan\delta =G^{\prime \prime }/G^{\prime }$ at the gel point: n=2δ/π [13]. Previously, we found n=0.51 [7] and use this value in the present study too. Note that, in the microrheology measurement, *n* corresponds to the exponent of mean-square displacement (MSD) of probe particles in the long-time region [30]. The value of *n* for this system is very close to the exponent for the Rouse mode (0.5). We previously distinguished these modes by demonstrating the time–cure superposition showing the change in direction of shifting the MSD curves at the gel point [7].

### 3.2. Rouse Relaxation Time $\tau_{R}$ of a Precursor Chain

There are several ways to estimate $\tau_{R}$. We consider the following four ways.

(*1*) According to Figure 2 in Ref. [7], the MSD of probe particles at $t\ll 10^{-4}s$ is almost independent of the cross-linker (Ni ion) concentration. This fact indicates that the MSD behavior observed in this short-time region reflects the segmental motion of precursor chain since the particle motion is independent of the extent of cross-linking. Thus, we see that $\tau_{R}$ is smaller than $10^{-4}s$.

(*2*) According to Ref. [31], the time required for each bond to rotate by overcoming the torsional energy barrier is estimated to be $\tau_{\mathrm{tor}}≅10^{-11}s$ . When the degree of polymerization of the precursor chain is $N_{p}≅10^{3}$ [7], the Rouse relaxation time is roughly estimated as $\tau_{R}≃\tau_{\mathrm{tor}}N_{p}^{2}≃10^{-5}s$ [31]. This value is consistent with that discussed in Label (*1*).

(*3*) A more accurate value of $\tau_{R}$ can be estimated by using the Rouse model [32] directly. For this purpose, we fitted the absolute modulus of the uncross-linked polymer solution (i.e., solution without cross-linkers) with the Rouse model whose complex modulus is given by

(29) $$\begin{array}{cc} G_{R}^{*}\left(\omega \right) & =G_{R}\sum_{j=1}^{\infty }\frac{i\omega j^{-2}\tau_{R}}{1+i\omega j^{-2}\tau_{R}}\\ & =\frac{G_{R}}{2}\left[\sqrt{i\omega \tau_{R}}\pi \coth\left(\sqrt{i\omega \tau_{R}}\pi \right)-1\right]. \end{array}$$

**Figure 2:**
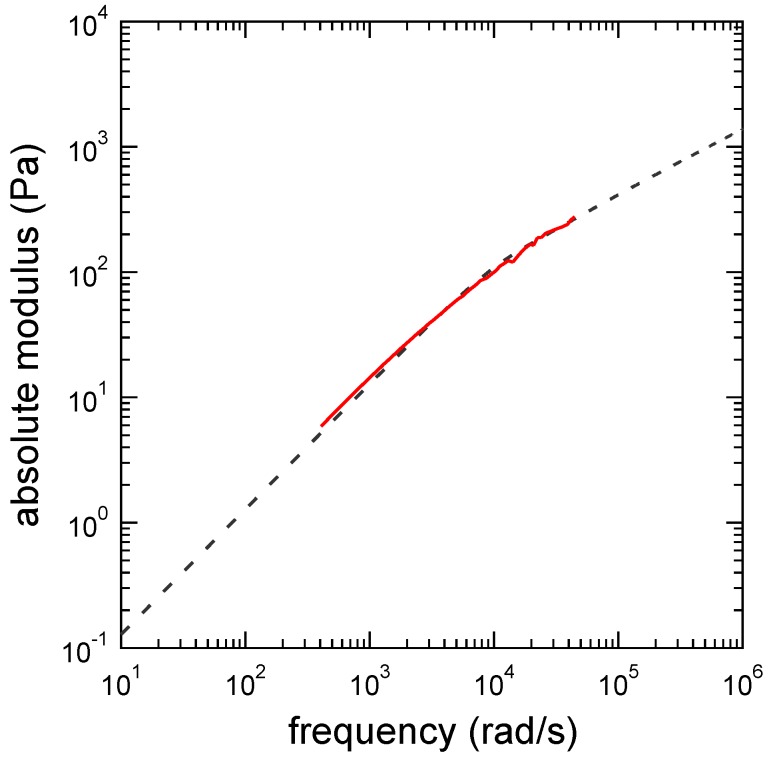
Absolute modulus of poly(acrylamide-*co*-vinylimidazole) (or PAAmVIm)/water system. The PAAmVIm concentration is $C_{p}=4\mathrm{wt}\%$. The solid and dashed lines indicate the absolute modulus of experimental and theoretical results, respectively.

Figure 2 shows the fitting result of the absolute modulus using Equation (29). It is noted that, in the fitting, we compared the absolute modulus from which the viscosity of the solvent η is subtracted (i.e., $|G^{*}\left(\omega \right)-i\omega \eta |$) with $|G_{R}^{*}\left(\omega \right)|$. From the fitting, we obtained $G_{R}=1.05\times 10^{2}\mathrm{Pa}$ and $\tau_{R}=7.40\times 10^{-5}s$. This $\tau_{R}$ value is also consistent with those discussed in Labels (*1*) and (*2*). The obtained $G_{R}$ is slightly lower than that estimated from the polymer chain density, i.e., $C_{p}RT/M$, where *R* and *M* are the gas constant and the molecular weight, respectively. The reason is still under investigation.

(*4*) Furthermore, $\tau_{R}$ corresponds to the time for the single polymer to diffuse its own radius of gyration $R_{g}$:

(30) $$\begin{array}{c} \tau_{R}≅\frac{R_{g}^{2}}{6D_{1}}, \end{array}$$

where $D_{1}$ is the diffusion coefficient of the single chain (see Equation 19). The friction of the polymer chain $\zeta_{1}$ can be expressed as the sum of the friction of each blob $\zeta_{0}=6\pi \eta \xi$:

(31) $$\begin{array}{c} \zeta_{1}=6\pi \eta \xi N, \end{array}$$

where η is the viscosity of the medium. We assume that the blobs diffuse in a medium having viscosity of water at $25^{\circ }C$ ($\eta =8.90\times 10^{-4}\mathrm{Pa}s$ [33]). According to the Einstein relation [26], the diffusion coefficient of the chain is

(32) $$\begin{array}{c} D_{1}≅\frac{k_{B}T}{\zeta_{1}}≅\frac{k_{B}T}{6\pi \eta \xi N}. \end{array}$$

From Equations (30) and (32), the longest Rouse relaxation time $\tau_{R}$ is given by

(33) $$\begin{array}{c} \tau_{R}≅\frac{R_{g}^{2}\xi \pi \eta N}{k_{B}T}. \end{array}$$

According to Equation (1), the size of blob ξ is expressed as $\xi =R_{g}N^{-1/2}$. Therefore, we obtain

(34) $$\begin{array}{c} \tau_{R}≅\frac{R_{g}^{3}\pi \eta N^{1/2}}{k_{B}T}. \end{array}$$

In the present study, the absolute temperature of the solution is T=298K [7]. The radius of gyration is already known from the static light scattering (SLS) measurement as $R_{g}=16\;nm$ [7]. If the number of blobs per chain *N* is changed between 1 to 1000, then $\tau_{R}$ takes a value between $3\times 10^{-6}s$ and $9\times 10^{-5}s$. These values are almost in the same order of magnitude with that obtained in Label (*3*), and also consistent with the values argued in Labels (*1*) and (*2*).

In the present analysis, we use the value obtained in Label (*3*), i.e., $\tau_{R}=7.40\times 10^{-5}s$.

### 3.3. Determination of the Other Parameters

**Figure 3:**
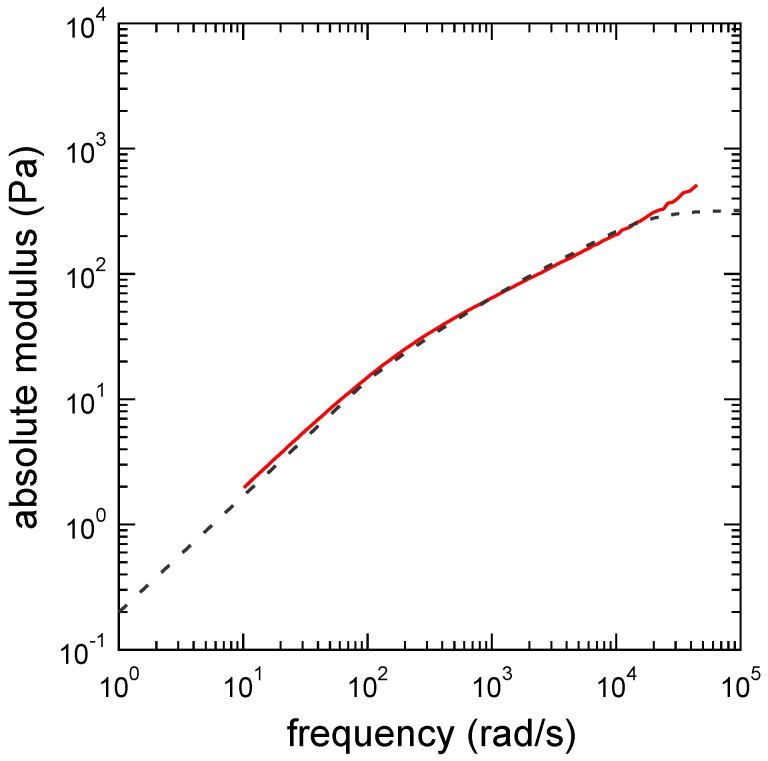
Absolute modulus of the PAAmVIm/Ni/water system at the gel point. The PAAmVIm concentration is $C_{p}=4\mathrm{wt}\%$ and the Ni ion concentration is $C_{\mathrm{Ni}}=1.1mM$. The solid and dashed lines indicate the absolute moduli of experimental and theoretical results, respectively.

**Figure 4:**
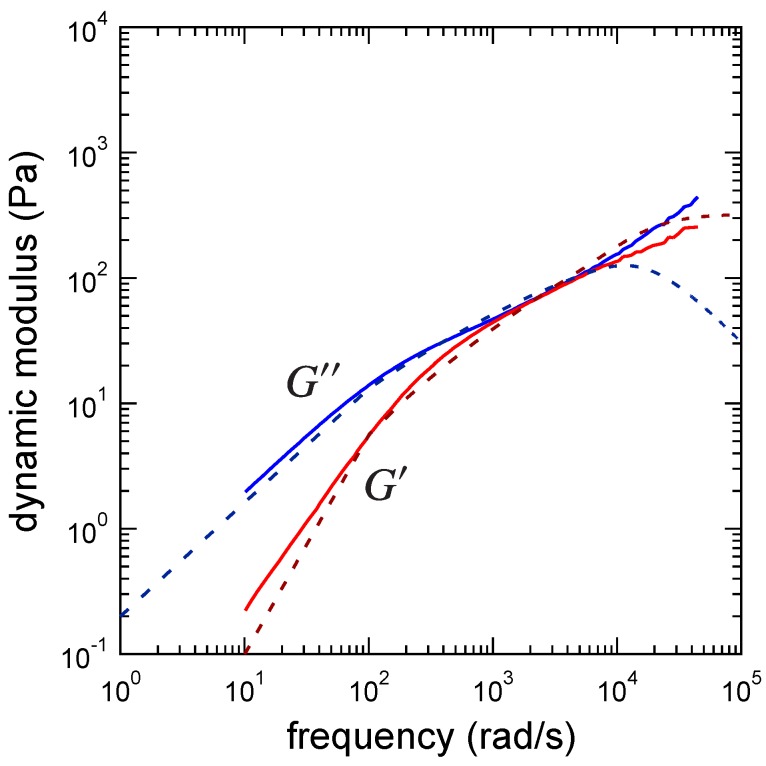
Dynamic modulus of the PAAmVIm/Ni/water system at the gel point where the PAAmVIm concentration is $C_{p}=4\mathrm{wt}\%$ and the Ni ion concentration is $C_{\mathrm{Ni}}=1.1mM$. The red and blue solid lines indicate the storage and loss moduli, respectively. The dashed lines indicate the storage and loss moduli from theory.

The other parameters cannot be directly determined from experiments. Thus, we used Equation (17) to fit the experimental result. Figure 3 and Figure 4 show the fitting results of the absolute modulus (Figure 3) and the dynamic modulus (Figure 4) of the PAAmVIm–Ni critical physical gel using Equation (17) with the fixed n=0.51 and $\tau_{R}=7.40\times 10^{-5}s$. From the fitting, we obtained $G_{0}=1.52\times 10^{2}\mathrm{Pa}$, α=2.69, and $m_{z}=9$. Thus, we see that the clusters consisting of nine polymer chains are the largest relaxed clusters. (We are assuming that the smallest unit that gives the shortest relaxation time is a precursor chain.). More detail about the largest relaxed cluster is discussed in Section 4.

Furthermore, using Equations (2), (22), (24), (27), and (28), we estimated the fractal dimension $d_{f}$ and the largest relaxed cluster size $R_{z}$. The results are shown in Table 2.

**Figure 5:**
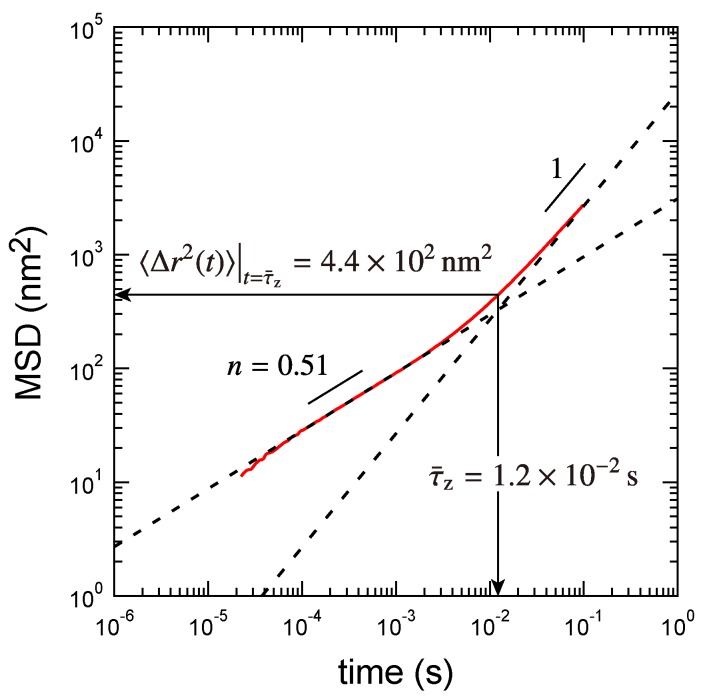
Mean-square displacement (MSD) of probe particles at the gel point (red solid line). The dashed lines are eye guides for the sub-diffusive regime with the exponent 0.51 and the diffusive regime.

The largest relaxed cluster size can also be estimated from the MSD of probe particles at the gel point as discussed in our previous study [7,20]. In the MSD at the gel point, a sub-diffusive regime (corresponding to the WC law) and a diffusive regime are observed in shorter and longer time regions, respectively (see Figure 5). A cross-over time between the two regimes is the apparent longest relaxation time ${\bar{\tau }}_{z}$. Within the time ${\bar{\tau }}_{z}$, the probe particle with the radius *R* diffuses the distance ${\left.\sqrt{\langle \Delta r^{2}\left(t\right)\rangle }\right|}_{t={\bar{\tau }}_{z}}$ and therefore the relation holds: ${\bar{\tau }}_{z}={\left.\langle \Delta r^{2}\left(t\right)\rangle \right|}_{t={\bar{\tau }}_{z}}R\pi \eta /k_{B}T$ [2,26]. On the other hand, the apparent longest relaxation time is comparable to the time required for the diffusion of the largest relaxed cluster over the distance $R_{z}$: ${\bar{\tau }}_{z}=R_{z}^{3}\pi \eta /k_{B}T$. From these two relations, the size of the largest relaxed cluster is obtained as

(35) $$\begin{array}{c} R_{z}=\sqrt[{3}]{{\left.\langle \Delta r^{2}\left(t\right)\rangle \right|}_{t={\bar{\tau }}_{z}}R}. \end{array}$$

For the PAAmVIm–Ni critical gel, the apparent longest relaxation time ${\bar{\tau }}_{z}$ and the MSD at ${\bar{\tau }}_{z}$ were estimated as ${\bar{\tau }}_{z}=1.2\times 10^{-2}s$ and ${\left.\langle \Delta r^{2}\left(t\right)\rangle \right|}_{t={\bar{\tau }}_{z}}=4.4\times 10^{2}\;nm^{2}$, respectively, by fitting the MSD of probe particles at the gel point (see Figure 5). As a result, the size of the largest relaxed cluster is obtained as $R_{z}=48\;nm$. This result is very close to that obtained when the hyperscaling hypothesis is assumed and the excluded-volume effect is screened ($R_{z}=49\;nm$).

The longest relaxation time of the system $\tau_{z}$ is governed by the largest relaxed cluster [6,20]. Meanwhile, the time obtained from the MSD of probe particles is the averaged relaxation time ${\bar{\tau }}_{z}$. For the PAAmVIm–Ni critical gel, we obtained $\tau_{z}=m_{z}^{\alpha }\tau_{R}=2.7\times 10^{-2}\;s$ and ${\bar{\tau }}_{z}=1.2\times 10^{-2}\;s,$ respectively, which is somewhat larger than that estimated from Equation (18): $4.1\times 10^{-3}\;s$. This is because the number of polymers composing the largest relaxed cluster $m_{z}$ in the present system is not so large, although we assumed a condition $m_{z}\gg 1$ in Equation (18). We also found that $\tau_{z}$ and ${\bar{\tau }}_{z}$ are not so separated, indicating that the distribution of clusters that contribute to rheological properties is narrow even at the gel point.

## 4. Discussion

For further understanding of the cluster structure, we discuss the results shown in Section 3 by comparing these results with the results of the PVA–borax critical physical gel reported in Ref. [20].

### 4.1. Comparison of the Experimental Conditions

To compare the experimental conditions of each system, we summarized the material properties in Table 3. The radius of gyration $R_{g}$ of the PAAmVIm chain is about two times larger than that of the PVA chain, although the weight average molecular weights $M_{w}$ (or DP’s) are almost the same. Since the polymer concentrations $C_{p}$ of the both systems are comparable, we see that the PAAmVIm–Ni system is above the overlap concentration, whereas the PVA–borax system is close to the overlap concentration. (The overlap concentrations $C_{p}^{*}$ of the PAAmVIm–Ni system and the PVA–borax system are estimated to be 1.0wt% and 7.2wt%, respectively. Here, $C_{p}^{*}$ was calculated by using the following equation: $C_{p}^{*}=3M_{w}/\left(4\pi R_{g}^{3}N_{A}\right)\times 10^{-4}\mathrm{wt}\%$, where $N_{A}$ is the Avogadro constant.)

The ability to cross-link is also different in both systems. The cross-linkable sites on PAAmVIm correspond to the imidazole groups (molar fraction in the feed: 0.1). Hence, the cross-linkable sites on the PAAmVIm chain are located at every 10 repeating units on average. On the other hand, for the PVA–borax system, the borate ions form complexes with adjacent OH functional groups on the PVA chains [20,34]. Therefore, the cross-linkable sites are located at any repeating unit on the chain, although the number of sites can be assumed to be half of the DP. Comparing these two conditions, we see that the number of cross-linkable sites on the PAAmVIm chain is approximately less than a fourth of that on the PVA chain. Meanwhile, the cross-linker concentrations $C_{c}^{*}$ at the gel point are 1.1mM (for the PAAmVIm–Ni system) and 3.4mM (for the PVA–borax system). Therefore, the number of cross-linkers per precursor chain ($≃C_{c}^{*}/C_{p}$) at the gel point of PAAmVIm–Ni system is approximately half of that of PVA–borax system.

**Figure 6:**
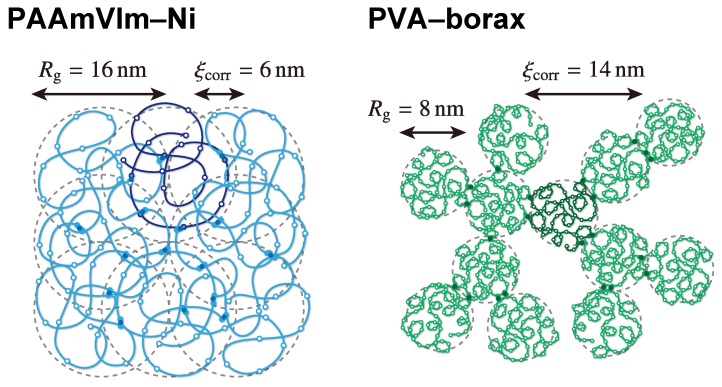
Schematic figures of the PAAmVIm–Ni cluster (**left**) and the PVA–borax cluster (**right**). In these figures, the differences of system properties, such as the radius of gyration $R_{g}$ of the precursor chain, the polymer concentration, the number of potentially cross-linkable sites on polymer chain, and the correlation length $\xi_{\mathrm{corr}}$ of concentration fluctuation are schematically emphasized. For clarity, one precursor chain is shown in different color in each figure. The cross-linkers involving in inter-molecular associations are also shown.

Schematic figures focusing on stoichiometric properties of these two systems are presented in Figure 6. The details are discussed in the next section.

### 4.2. Comparison of the Parameters

The parameters $G_{0}$, $\tau_{R}$, α, *n*, and $m_{z}$ of each system are compared in Table 4.

Since $G_{0}$ of the PAAmVIm–Ni system is larger than that of the PVA–borax system, we can guess that the cross-linking density of PAAmVIm–Ni system is lower than that of the PVA–borax system. Considering that the polymer concentrations are roughly comparable, it is expected that the PAAmVIm precursor chains are cross-linked more sparsely. This condition makes $m_{z}$ and $\tau_{z}$ of the PAAmVIm–Ni system small, although the activation energy of the PAAmVIm–Ni coordination bond is larger than that of the PVA–borate ion complexation (see Table 3).

The Rouse relaxation time of the precursor chain $\tau_{R}$ of the PAAmVIm–Ni system is larger than that of the PVA–borax system. This is a reasonable result considering that the PAAmVIm chains are more overlapped than the PVA chains and therefore the mobility of the PAAmVIm chain is more suppressed. The collective diffusion modes were observed by the DLS measurements in both systems, and the correlation lengths were 6 nm (for the PAAmVIm–Ni system) and 14 nm (for the PVA–borax system) (see Table 3) [7,20]. This mode is attributed to collective fluctuations of the entangled or cross-linked polymer chains, and therefore this result supports the existence of the contact between PAAmVIm chains.

In Table 4, the true relaxation time $\tau_{z}$ and the apparent relaxation time ${\bar{\tau }}_{z}$ of each system are also compared. The difference between $\tau_{z}$ and ${\bar{\tau }}_{z}$ in the PAAmVIm–Ni system is 10 times smaller than that in the PVA–borax system. This result indicates that the distribution of clusters that contributes to rheological properties of the PAAmVIm–Ni system is narrower than that of the PVA–borax system.

Using the parameters shown in Table 4, we can obtain the fractal dimension $d_{f}$ and the size of the largest relaxed clusters $R_{z}$. The results are shown in Table 5. The value of $R_{z}$ that is close to that estimated from the MSD of probe particles (see Equation (35)) is highlighted in bold italic for each system. We can guess that the excluded-volume effect is unscreened for the PVA–borax system, while it is screened for the PAAmVIm–Ni system (the hyperscaling hypothesis is assumed for both systems). This result on the excluded-volume effect as well as the value of the corresponding fractal dimension seems reasonable for the following reasons. It is widely accepted that the excluded-volume effect is gradually screened as the polymer concentration increases because the fluctuation of polymer density decreases [2,27,28]. In Section 4.1, we showed that the studied polymer concentration of the PAAmVIm–Ni system is higher than the overlap concentration, whereas that of the PVA–borax system is near the overlap concentration. In addition, the value of the correlation length of the collective diffusion mode indicates that the concentration fluctuation of PAAmVIm–Ni system is smaller than that of the PVA–borax system (see Figure 6). Considering these results, the excluded-volume effect in the PAAmVIm–Ni system should be more screened than that in the PVA–borax system. Furthermore, we found that $d_{f}$ of the PAAmVIm–Ni system is smaller than that of the PVA–borax system, indicating that the PAAmVIm–Ni cluster has a sparser structure than the PVA–borax cluster. That is, the PAAmVIm precursor chains having a large pervaded volume are weakly cross-linked by fewer cross-linkers (see Figure 6).

## 5. Conclusions

In this article, we studied the viscoelasticity of critical physical hydrogel from a theoretical viewpoint. Our physical hydrogel consists of polyacrylamide-derivative associating polymers containing imidazole groups (PAAmVIm) and Ni ion [7]. The viscoelastic quantities of physical gel do not diverge at the gel point due to the finite lifetime of physical cross-links. The viscoelastic behavior in the vicinity of physical gel point is characterized by the largest cluster, which can relax before it breaks [6]. In the present study, we estimated the size of the largest relaxed cluster using the phenomenological theory to describe the viscoelastic properties of critical physical gels [20,22].

We found that the largest relaxed cluster of the PAAmVIm–Ni system is composed of nine precursor chains. When the hyperscaling hypothesis is assumed and the excluded-volume effect is screened, the fractal dimension $d_{f}$ and the size $R_{z}$ are, respectively, $d_{f}=2.0$ and $R_{z}=49\;nm$. The obtained value of $R_{z}$ is comparable with that previously estimated from the mean-square displacement of probe particles embedded in the sample at the gel point in microrheology [7].

We also discussed the cluster structure of the PAAmVIm–Ni system by comparing it with that of the PVA–borax system [20]. The largest relaxed cluster of the PAAmVIm–Ni system is smaller than that of the PVA–borax system, indicating that the connection between PAAmVIm precursor chains is weaker than that between PVA chains. In addition, the fractal dimension of the PAAmVIm–Ni system is smaller than that of the PVA–borax system, that is, the PAAmVIm–Ni cluster has a sparser structure than the PVA–borax cluster. This result indicates that the cluster is formed by the weakly cross-linked polymer chains having a large pervaded volume.

## Figures and Tables

**Table 1:** Fractal dimensions for four cases, depending on whether the excluded-volume effect is unscreened or screened and whether the hyperscaling hypothesis is assumed or not.

Hyperscaling Hypothesis Excluded-Volume Effect	Not Assumed		Assumed	
	Unscreened	Screened	Unscreened	Screened
Fractal dimension $d_{f}$	Equation (22)	Equation (24)	Equation (27)	Equation (28)
Index (used in Table 2)	(I)	(II)	(III)	(IV)

**Table 2:** Estimated fractal dimension $d_{f}$ and size of the largest relaxed cluster $R_{z}$. $R_{z}$ was calculated from Equation (2) by putting $m=m_{z}=9$.

Hyperscaling Hypothesis Excluded-Volume Effect	Not Assumed		Assumed	
	Unscreened	Screened	Unscreened	Screened
Index (see Table 1)	(I)	(II)	(III)	(IV)
Fractal dimension $d_{f}$	1.2	1.4	3.9	2.0
Largest relaxed cluster size $R_{z}\left(nm\right)$	1.0 × 10^2^	82	28	49

**Table 3:** Comparison of properties of precursor chains and cross-linkers in the PAAmVIm– Ni system and in the PVA–borax system. ${}^{†}$ $M_{w}$ of PAAmVIm is obtained by SLS measurement, where the refractive index increment is dn/dc = 0.146 mL $g^{-1}$ [35]. ${}^{‡}$ DP of the PAAmVIm chain was calculated by regarding the repeating unit as acrylamide. ${}^{§}$ $R_{g}$ of the PAAmVIm chain was experimentally obtained by SLS measurement, whereas that of the PVA chain was calculated using the Gaussian chain assumption [7,20]. ${}^{∥}$ $\xi_{\mathrm{corr}}$ is the correlation length of the collective diffusion mode observed in the DLS measurement in each system [7,20].

System	PAAmVIm–Ni	PVA–borax
precursor polymer	**PAAmVIm**	**PVA**
polymer concentration ($C_{p}$)	4 wt %	5.5 wt %
weight average molecular weight ($M_{w}$) ${}^{†}$	1.7 × 10^5^ g mol^−1^	8.9 × 10^4^ g/mol
degree of polymerization (DP) ${}^{‡}$	2.4 × 10^3^	2.0 × 10^3^
radius of gyration ($R_{g}$) ${}^{§}$	16 nm	7.9nm
number of potentially cross-linkable sites per polymer chain	2.4 × 10^2^	1.0 × 10^3^
fraction of potentially cross-linkable sites	1/10	1/2
cross-linker	**Ni ion (Ni2+)**	**borate ion (B(OH)^−^_4_)**
cross-linker concentration at the gel point ($C_{c}^{*}$)	1.1 mM	3.4 mM
correlation length ($\xi_{\mathrm{corr}}$) ${}^{∥}$	6 nm	14 nm
activation energy	86 kJ mol^−1^	42 kJ mol^−1^

**Table 4:** Comparison of the parameters $G_{0}$, $\tau_{R}$, α, *n*, and $m_{z}$ between the PAAmVIm–Ni system and the PVA–borax system. The true relaxation time $\tau_{z}$ and the apparent relaxation time ${\bar{\tau }}_{z}$ are also listed as a reference. ${}^{†}$ $\tau_{R}$ of the PAAmVIm–Ni system was estimated in the uncross-linked system (see Section 3.2), whereas that of the PVA–borax system was estimated in the presence of borate ions [20]. ${}^{‡}$ *n*s of both systems were determined from the loss tangent at the gel point. The other parameters were obtained by fitting the absolute modulus of the critical gel used with Equation (17).

System	$G_{0}\left[\mathrm{Pa}\right]$	$\tau_{R}\left[s\right]$ ${}^{†}$	α	$n^{‡}$	$m_{z}$	$\tau_{z}\left[s\right]$	${\bar{\tau }}_{z}\left[s\right]$
**PAAmVIm–Ni**	1.5 × 10^2^	7.4 × 10^−5^	2.69	0.5	9	2.7 × 10^−2^	1.2 × 10^−2^
**PVA–borax**	1.7 × 10^3^	5.9 × 10^−7^	1.95	0.59	550	1.3 × 10^−1^	1.3 × 10^−2^

**Table 5:** The fractal dimensions $d_{f}$ and the sizes of the largest relaxed cluster $R_{z}$ of the PAAmVIm–Ni system and the PVA–borax system. In the table, $R_{z}$ which is close to that obtained from the MSD of the probe particles, is highlighted in bold italic for each system.

	Hyperscaling Hypothesis Excluded-Volume Effect	Not Assumed		Assumed	
System		Unscreened	Screened	Unscreened	Screened
**PAAmVIm–Ni**	$d_{f}$	1.2	1.4	3.9	2.0
	$R_{z}\left[nm\right]$	1.0 × 10^2^	82	28	***49***
**PVA–borax**	$d_{f}$	2.1	1.7	3.1	1.9
	$R_{z}\left[nm\right]$	1.6 × 10^2^	3.3 × 10^2^	***61***	2.2 × 10^2^

## Abbreviations

The following abbreviations are used in this manuscript:

DLS	Dynamic Light Scattering
DWS	Diffusing-Wave Spectroscopy
PAAmVIm	Poly(AcrylAmide-*co*-1-VinylImidazole)
	[Polyacrylamide-derivative associating polymers containing imidazole groups]
PVA	Poly(Vinyl Alcohol)
SLS	Static Light Scattering
WC	Winter–Chambon
